# REIMAGINING DISASTER AND EMERGENCY PREPAREDNESS IN LONG-TERM CARE: EXAMPLES FROM FLORIDA AND TEXAS

**DOI:** 10.1093/geroni/igac059.1376

**Published:** 2022-12-20

**Authors:** Lindsay Peterson, Ross Andel, Sue Anne Bell

## Abstract

A thread common to all disasters is the effect on human health that results when the health care infrastructure and delivery of health services are disrupted. This view has reinforced the importance of an all “all-hazards approach” to preparedness, whereby disaster response planning incorporates principles common to all events and can be adapted to specific contingencies. Preparing for and responding to a disaster in long-term care (LTC) requires a broad view of multiple events that can disrupt daily life and needed services for LTC residents (e.g. hurricanes, pandemics). This symposium will examine the effects of varied emergency events on older adults residing in nursing homes (NHs) and assisted living communities (ALCs) using quantitative and qualitative methodologies. The first presentation will discuss morbidity and mortality of NH residents exposed to Hurricane Harvey (Texas). The second is an investigation of NH direct care staffing during the recent Winter Storm Uri (Texas). The third presentation qualitatively explores the challenges of providing care to residents living with dementia during the COVID-19 pandemic, based on interviews with Florida NH and ALC administrators. The fourth quantitatively and qualitatively explores issues related to resident-to-resident contact restrictions in Florida ALCs. Finally, we will discuss the application of the 4Ms Age Friendly framework (What Matters, Medication, Mentation, and Mobility) to disaster preparedness in LTC. This symposium will provide information that can be used to develop or revise public policies to improve preparedness for and response to a range of emergency events in NHs and ALCs.

